# Immunogenomics: a foundation for intelligent immune design

**DOI:** 10.1186/s13073-015-0247-y

**Published:** 2015-11-19

**Authors:** Robert A. Holt

**Affiliations:** Canada’s Michael Smith Genome Sciences Centre, BC Cancer Agency, Vancouver, British Columbia V5Z 1L3 Canada; Department of Molecular Biology & Biochemistry, Simon Fraser University, Burnaby, British Columbia V5A 1S6 Canada; Department of Medical Genetics, University of British Columbia, Vancouver, British Columbia V6T 1Z4 Canada

## Abstract

The complexity of the immune system is now being interrogated using methodologies that generate extensive multi-dimensional data. Effective collection, integration and interpretation of these data remain difficult, but overcoming these important challenges will provide new insights into immune function and opportunities for the rational design of new immune interventions.

## Immunogenomics is an information science

Just by counting, it becomes clear that the adaptive immune system is the biggest source of human genetic variation. Each of us carries four to five million single nucleotide polymorphisms, and the *HLA* locus, the chromosomal region most dedicated to distinguishing self from non-self, contributes more to this total than any other part of our genome [1]. Adding, for each of us, the millions of uniquely randomized T- and B-cell receptor genes that encode our immune repertoires, it becomes apparent that at the level of DNA, immunogenomic profiles are what make us most unique. This diversity is the source of the genetic plasticity that allows us to thrive as individuals and as a species in an environment of persistent yet unpredictable immune challenge.

Immunogenomics, however, is not actuarial science — it is an information science. It is a broad and diversified field that has a long history. With advancing technology, we continue to build on the hard work and remarkable insights that established the fundamental principles and mechanistic underpinnings of the immune system, such as somatic recombination, clonal selection and self-tolerance — ideas that when first described must have seemed too outlandish to be real. Next-generation sequencing is clearly playing a transformative role in immunogenomics research, as in many areas of the life sciences, which makes this special issue on ‘Immunogenomics in health and disease’ very timely. Other advancing technologies are equally impactful; for example, mass cytometry can now provide an incredibly nuanced view of the phenotypic diversity among immune cell subsets. Nevertheless, working across technology platforms remains a challenge. It is not immediately obvious how to best interrogate billions of sequences from cell populations defined by hundreds of markers, derived from individuals with unique genetic backgrounds and personalized histories of immune exposure. Standardized laboratory workflows, data formats, experimental designs and statistical methods will be necessary and, when available, will likely place immunology among the biggest of ‘Big Data’ enterprises in the life sciences. Here again, we benefit from our scientific predecessors, who did not shy from the difficult task of annotating the immune system, and who developed an immune ontology [2, 3] that continues to serve as a very important base in this new era.

## What do we hope to discover?

Will advances in immunogenomics reinforce current views, asymptotically filling smaller gaps in our knowledge with more and more prodigious data, or will immunogenomics be transformative? One can’t know in advance, but this discipline is now well positioned to shed light on both new and long-standing questions. For example, mapping the interactions between the microbiome and host immunity that determine commensal versus adversarial relationships is a new challenge, and this work has just begun in earnest. A more enduring gap in our knowledge is the very incomplete view we have of allelic variation within immune receptor genes, a gap that persists due to the structural complexity of these loci and the tendency of investigators to focus their attention on somatic rather than germline variation. An effort to provide, in particular, a more comprehensive view of B-cell receptor (BCR) alleles will greatly facilitate the interpretation of antibody repertoire data, and will in turn facilitate therapeutic antibody development, by making allelic variants more readily distinguishable from somatic hypermutations that allow for its detection.

In the realm of cellular immunity, the determinants of T-cell lineage specification are being elucidated, but it remains unclear how rigidly immune cell phenotypes are maintained. This is of key importance for T cells, given that immunoreactivity may be either activated or inhibited depending on the subset. Likewise, the rules of immunodominance, whereby response to a given antigen is a function of other antigens present, remains opaque. Perhaps most concerning, however, is the persistence of the notion that T cells are antigen specific. It is true that in isolation a given T cell can be shown to interact selectively with a major histocompatibility complex (MHC) presenting one peptide but not another, but the ‘one T cell–one antigen’ view articulated in early formulations of the clonal selection theory has been thoroughly refuted on a theoretical basis (the millions of T-cell clonotypes each of us maintains, if monospecific, could not protect against encounters with more than 10^15^ potential peptide antigens) and by direct observation of polyspecificity in experimental systems [4].

It is inconvenient to have to consider promiscuity when developing T-cell therapeutics, and this issue tends to be ignored for the simple reason that we are not yet capable of routinely measuring it. This is particularly relevant to cancer therapy where the strategy of stimulating anti-tumor immunity by blocking inhibitory immunological checkpoints, which hold back otherwise reactive T cells, has shown remarkable success [5]. It is not yet possible, however, to predict who will respond to these therapies or the severity of the side effects because we cannot yet determine the antigen specificities of the T cells that will be unleashed, or the consequences of their cross-reactivities. Further, it tends to be assumed that the T cells awoken by immune checkpoint blockade are originally activated by tumor antigens, but then become dormant. It is possible, however, that the anti-cancer effects of tumor-resident T cells are incidental, and represent fortuitous recognition of tumor antigens by broadly cross-reactive T cells. This is a speculative view, but one that needs further consideration. It is consistent with the observation of viral-specific T cells in the tumor environment, with the intriguing (but as yet unreplicated) finding of microbial signatures being prevalent in the neo-antigen repertoires of patients responding to checkpoint blockade [6], and with established precedents of heterologous immunity [7].

## Applying what we learn

Approaching immunogenomics as information science, in pursuit of an increasingly comprehensive view of connectivity within the immune system at rest and under challenge, is likely to lead to new and better strategies for immune intervention. For example, if T-cell promiscuity does prove to be an important factor underlying the efficacy of cancer immunotherapy, then the design of any treatment that leverages natural T-cell reactivity should take this into account. Efforts to improve adoptive cell therapies may be best focused on shaping the on-target versus off-target properties of T cells so they can be exploited as broadly reactive agents, while redoubling efforts in therapeutic antibody and chimeric antigen receptor development for therapeutic applications that require laser-like target specificity.

Another area where new insights from immunogenomics might have medical relevance is in immune regeneration. Eventually, we all face the certainty of immune decline. Immunosenescence is characterized by dwindling production of naïve lymphocytes due to myeloid skewing and thymic degeneration, by increasing representation of functionally and proliferatively exhausted memory cells, and by deficiencies in innate immune mechanisms [8–10]. Immune decline, already underway as we emerge from adolescence, is an underlying factor in a wide spectrum of age-related disorders and a key challenge for regenerative medicine. Will engineered immunity be part of the solution? The derivation of induced pluripotent stem cells (iPSCs) from peripheral T cells is now routine, and the feasibility of re-differentiating iPSCs of T-cell origin into rejuvenated naïve effector cells that maintain antigen specificity but show renewed proliferative capacity has now been demonstrated [11, 12]. This illuminates a path to manufactured, heterochthonous immunity that has the potential to outperform substantially the current vaccine paradigm that fails the elderly and immunocompromised. If individual T-cell clones can be rejuvenated in this manner, why not B cells too? Why not ensembles of lymphocytes with defined specificities that can be rejuvenated and released in their host as protective anti-pathogen or anti-tumor swarms? It’s still early days, but advancing technologies and creative immunogenomic approaches are providing an increasingly detailed view of how immunity is orchestrated. With the roles and dependencies of the various players coming into clearer focus, and the tools on hand to manipulate them, a future of intelligent immune design awaits.

## Abbreviation

iPSC: induced pluripotent stem cell
